# Epidemiologic Features of Enterovirus 71-Associated Hand-Foot-and-Mouth Disease from 2009 to 2013 in Zhejiang, China

**DOI:** 10.3390/ijerph14010033

**Published:** 2016-12-30

**Authors:** Zhifang Wang, Huakun Lv, Wenming Zhu, Zhe Mo, Guangming Mao, Xiaofeng Wang, Xiaoming Lou, Yongdi Chen

**Affiliations:** 1 Department of Immunization Programme, Zhejiang Provincial Center for Disease Control and Prevention, 3399 Binsheng Road, Hangzhou 310051, China; zfwang@cdc.zj.cn (Z.W.); hklv@cdc.zj.cn (H.L.); 2 Department of Environmental and Occupational Health, Zhejiang Provincial Center for Disease Control and Prevention, 3399 Binsheng Road, Hangzhou 310051, China; wmzhu@cdc.zj.cn (W.Z.); zhmo@cdc.zj.cn (Z.M.); 3 Key Medical Research Center, Zhejiang Provincial Center for Disease Control and Prevention, 3399 Binsheng Road, Hangzhou 310051, China; 4 Department of Science and Technology Information, Zhejiang Provincial Center for Disease Control and Prevention, 3399 Binsheng Road, Hangzhou 310051, China

**Keywords:** enterovirus 71, hand-foot-and-mouth disease, epidemiology, surveillance

## Abstract

Enterovirus 71 (EV71) usually causes hand-foot-and-mouth disease (HFMD) with severe clinical symptoms and even deaths in China. There is no efficient antiviral drug to protect against severe EV71-associated HFMD, making the development of EV71 vaccines therefore a priority. However, the potential target subject population(s) to be immunized with EV71 vaccine are not well understood. In this study, we characterized the epidemiology regarding EV71-associated HFMD on the basis of provincial-level surveillance. We extracted data on EV71-associated HFMD from the National Notifiable Disease Reporting System in Zhejiang Province, China between 1 January 2009 and 31 December 2013 (*n* = 7650). The higher incidence rate of EV71 cases occurred in those children aged 12–23 months, with boys being predominant. Interestingly, different peaks activities of EV71 infection was observed in different calendar year, with one peak in 2009 and 2013 and two peaks in 2010–2012. However, EV71 infection seemed to predominately occur in warm season and a distinguished cyclic peak that seemed to be of about 12 months. Children aged 12–23 months are thus identified as an important target population for public health intervention, for example, it is recommended that these key subjects immunized with EV71 vaccine. In addition, an enhanced surveillance system for EV71-associated with HFMD needs to focus on generic and phylogenetic analysis.

## 1. Introduction

Hand-foot-and-mouth disease (HFMD) is an emerging infectious disease caused by a group of viruses, including enteroviruses, coxsackieviruses, echoviruses, and polioviruses. Infection with enterovirus 71 (EV71) is of particular concern as it accounts for a major proportion of severe diseases and deaths in infants and young children during large scale HFMD outbreaks, particularly in the Asia-Pacific region [1,2,3,4,5].

EV71-associated HFMD, which causes fever, skin rash, blisters and ulcers on hands, feet and mouth, and may further develop into pulmonary oedema, aseptic meningitis, encephalitis of the brainstem, and other complications, has become an important public health issue in China in recent years [6]. National notifiable diseases surveillance on EV71-associated HFMD showed that this disease had a high incidence rate (1.2 cases per 1000 person-years) and accounted for approximately 500–900 reported deaths per year in China from 2008 through 2012 [7].

To date, there is no efficient antiviral drug to protect against severe EV71-associated HFMD. Moreover, health education and social distancing measures seem not to have a significant effect on controlling and preventing EV71-associated HFMD [8]. The development of EV71 vaccines therefore is a priority. Recently, several EV71 vaccine candidates were evaluated in humans in mainland China, Singapore, and Taiwan [9,10,11,12]. Some are expected to be licensed in the near future. The study of epidemiological characteristics is critical to provide the baseline data for future vaccine clinical trials. Therefore, this study will focus on the epidemiological study of EV71-associated HFMD in Zhejiang Province based on the surveillance data from the National Notifiable Disease Reporting System (NNDRS).

## 2. Subjects and Methods

### 2.1. Sample Collection

From 1 January 2009 to 31 December 2013, samples were collected from the first five patients with probable disease who visited outpatient departments every month in each of 90 counties or districts throughout the whole province of Zhejiang. The samples from all severe cases were also collected.

### 2.2. Case Definitions

HFMD is a class “C” notifiable disease in China. Physicians in hospitals are required to report infected cases into the NNDRS within 24 h. All probable and laboratory-confirmed HFMD cases have been reported on a mandatory basis since 2 May 2008. A probable case of HFMD is diagnosed as a patient with popular or vesicular rash on hands, feet, mouth or buttocks, with or without fever. A laboratory-confirmed case was defined as a probable case with a laboratory evidence of infection detected by polymerase chain reaction (PCR) or virus isolation, including EV71, COX 16, as well as non-EV71 and non COX16. Cases were classified as severe if they had any neurological complications or cardiopulmonary complications.

### 2.3. Reported EV71-Associated HFMD

All laboratory-confirmed cases of EV71-associated HFMD are reported. The epidemiological information of each EV71-associated HFMD case was included and reported as follows: demographic information (gender, birth date, occupation, symptoms onset date, and diagnosis date), and virus serotype (EV71) for laboratory-confirmed cases.

### 2.4. Data Resources and Data Analysis

Reported EV71-associated HFMD cases during 1 January 2009 and 31 December 2013 were exported from the NNDRS. Data on demographic information during the study period were exported from annual statistical review reports published by the Zhejiang Provincial Bureau of Statistics [13]. Age groups were defined as 0–11, 12–23, 24–35, 36–47, 48–59, 60–71, and ≥72 months. Occupations were reported according to internet-based NNDRS user guide, including scattered children, preschool children, school children, and others. Scattered children are defined as children whose care were given by their family members. Preschool children are classified as those children who are enrolled in the kindergarten or the nursery. School children are classified as those enrolled in the elementary school and above. The geographic distribution of reported EV71 cases was determined on the basis of the 11 cities of Zhejiang Province.

The reported incidence rate (R) of EV71-associated HFMD cases was calculated by dividing the number of reported EV71-associated HFMD cases (C) via the NNDRS by the number of inhabitants (I) registered in local public health facilities (R = C/I). Reported incidence rates were calculated per 100,000 persons. The incidence rates, stratified by the overall population, gender, and age group were adjusted according to the 2013 Zhejiang provincial population distribution using the direct method of standardization [14]. Population density is calculated by dividing the number of inhabitants registered in public health facilities by land area in square kilometers. Cases density is calculated by dividing the number of reported cases by land area in square kilometers. Person’s correlation test was used to analyze whether population density and cases density correlated with each other. All calculations were performed using Statistical Package for the Social Sciences, version 16.0 (SPSS, Chicago, IL, USA) and Excel 2016 (Microsoft, Redmond, WA, USA).

The ethics committee of the Zhejiang Provincial Center for Disease Control and Prevention approved this study (Ethical approval code: ZJCDC20140224). Because data were acquired from the secondary hand and analyzed anonymously, no participant was required to provide written informed consent.

## 3. Results

### 3.1. Characteristics of Severe and Fatal EV71-associated HFMD Cases

Between 2009 and 2013, a total of 526,446 HFMD cases were reported via NNDRS, including 508,469 (96.6%) probable cases and 17,977 (3.4%) laboratory-confirmed cases. Among the total of 17,977 reported laboratory-confirmed HFMD cases, 7650 (42.6%) cases were reported to be infected with EV71 virus, 4254 (23.7%) cases were associated with Cox 16 virus and 6073 (33.8%) were other non-EV71 and non-Cox16 viruses. Of the 7650 cases, 909 (11.9%) cases were severe and 76 cases (1.0%) resulted in death of the patient, with the highest incidence rate corresponding to the 12–23 months and 0–11 months age groups, respectively, between 2009 and 2013 (Table S1). Of 909 severe cases, 615 cases (66.6%) were males and 294 (32.3%) were females. The severity rate of males-to-females is 2.0. Among the 76 fatal cases, 45 (59.2%) were males and 31 (40.8%) were females. The fatality rate of males-to-females is 1.5.

### 3.2. Description of Overall Trends of EV71-Associated HFMD

**Figure 1:**
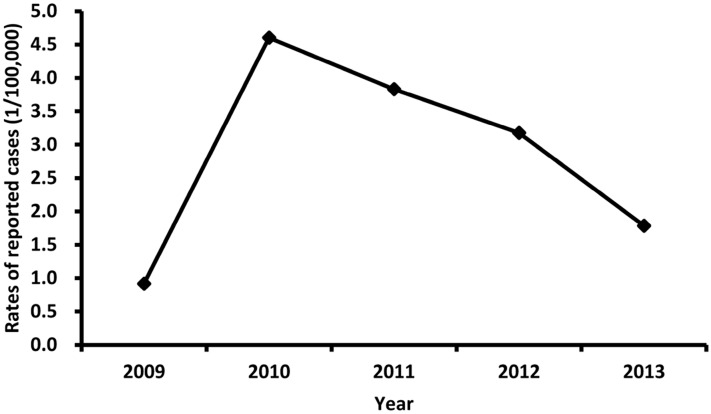
Incidence rate of reported EV71 cases by year, 2009–2013.

The annual average incidence rate of reported HFMD cases with EV71 infection was 2.9 cases per 100,000 persons between 2009 and 2013. The incidence rate maintained a low level in 2009 (0.9 cases per 100,000 population). It was sharply increased to peak in 2010 (4.6 cases per 100,000 population) and gradually decreased from 3.8 cases per 100,000 population in 2011 to 1.8 per 100,000 in 2013 (Figure 1).

### 3.3. Age Distribution

Incidence rate of EV71-associated HFMD cases varied greatly with age group during the study period. The highest incidence rate occurred in children aged 12 months to 23 months, followed by the rates in children aged 24–35 months and those aged 36–47 months, respectively. The lowest rate in those population aged ≥72 months (Table 1).

**Figure 2:**
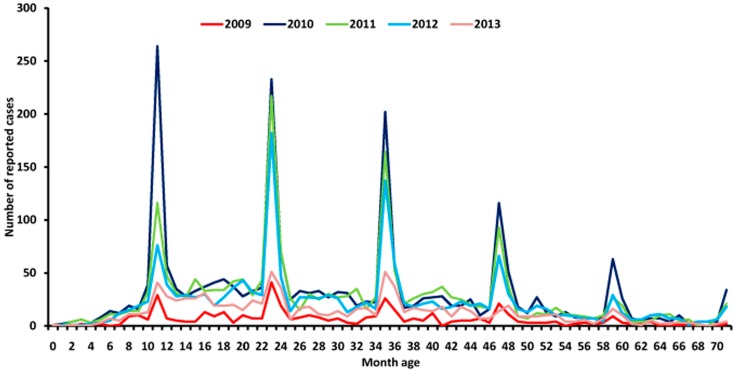
Number of reported EV71 cases aged less than 71 months by one month age, 2009–2013.

Since children younger than 71 months accounted for 97% of the total reported cases (Figure S1), they were chosen to further analysis the yearly trend of the number of reported cases per month age group between 2009 and 2013 (Figure 2). The peaks and troughs of these yearly trends matched very well with each other. It was observed that peaks occurred among children aged 11 months, 23 months, 35 months, 47 months, 59 months, and 71 months, respectively. The height of peaks however steadily deceased with month age.

### 3.4. Gender Distribution

The incidence rates of reported EV71 cases by gender are presented in Table 2. For each year between 2009 and 2013, the rate was significantly higher in males than females. During the study period, 4794 cases were males and 2856 were females, correspondingly, the incidence rates of reported EV71 cases were 3.5 cases per 100,000 males and 2.2 cases per 100,000 females (Table 2). The incidence ratio of boy-to-girl was 1.7:1 in 2009, 1.6:1 between 2010 and 2012, and 1.4:1 in 2013, respectively.

### 3.5. Occupational Distribution

During 2009–2013, the majority of EV71 cases occurred in scattered children (Table 3). Total of 5223 cases (68.3%) of EV71 infections were from scattered children, 2256 cases (29.5%) were from preschool children and 155 cases (2.0%) were school children.

### 3.6. Seasonal Distribution

**Figure 3:**
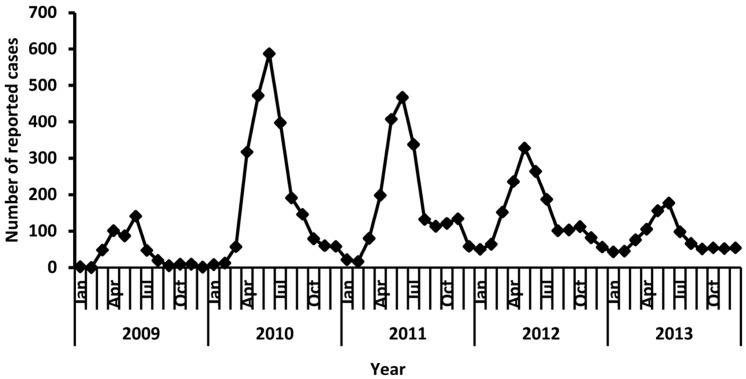
Monthly distribution of reported EV71 cases, 2009–2013.

Cases of EV71 infection were reported throughout the year during 2009–2013, but its annual predominance appeared in different month period. Figure 3 showed different peak activities of EV71 infection between 2009 and 2013. One peak was observed in 2009 and 2013 while two peaks were observed in 2010, 2011 and 2012. A small peak (from April to June) was seen in 2009 and 2013, while a high peak (from April to August) and a second, small peak (around October) were found in 2010, 2011 and 2012.

A bigger reported number of EV71 cases (≥500 cases) were observed in warm season (from May to October) between 2009 and 2013, compared with the reported number in cold season (from January to April, and from November to December) (Figure 3).

### 3.7. Geographic Distribution

**Figure 4:**
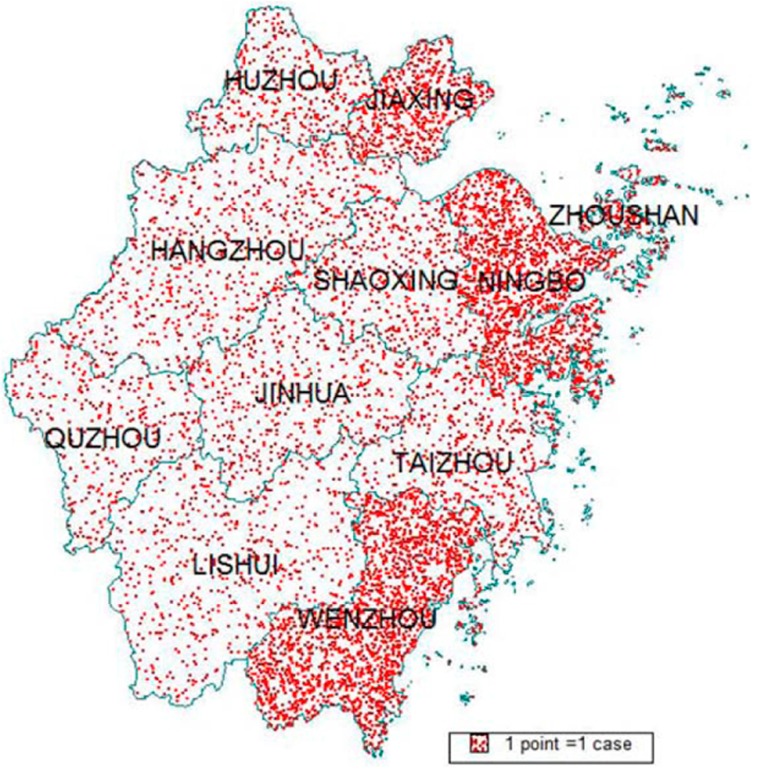
Geographic distribution of accumulated reported EV71 cases, 2009–2013.

Figure 4 shows the geographic distribution of accumulated reported EV71 cases from 2009 through 2013. Cases infected with EV71 were reported in 11 cities throughout the whole province, with the high reported number in Wenzhou, Ningbo, Jiaxing, and Zhoushan. The top four cities in terms of the number of reported EV71 cases per square kilometer were Wenzhou, Ningbo, Jiaxing, and Zhoushan, respectively (Table 4). Cases density and population density were correlated with each other (Pearson’s correlation coefficient = 0.80, *p* = 0.003).

## 4. Discussion

To the best of our knowledge, this is the first population-based study on epidemiological description of EV71-associated HFMD in Zhejiang Province, China, covering 7650 cases from the NNDRS in the past 5 years. The surveillance results showed the estimated annual incidence rate of EV71-assoicated HFMD was similar as the annual average incidence rate of EV71-associated HFMD of the whole nation (5/100,000) [7].

The trends of incidence rate of reported EV71-associated HFMD cases by age appeared upward and then downward, with a low incidence rate occurred in children aged 0–11 months and in those aged ≥60 months and a peak incidence were seen in children aged 12–23 months. This surveillance results indicated that children aged 0–23 months were more susceptible to develop severe clinical symptoms, even death. These findings were similar with the previous studies [7,15,16,17,18]. The potential reason for the age-specific incidence trend may be related with pre-existing antibodies against EV71. Previous seroepidemiological studies showed that the trends of EV71 neutralizing antibodies during early childhood were changed as a “V” shape with increased age. For neonates, most of them had maternal serological antibodies. However, their maternal EV71 neutralizing antibodies levels waned when their month age increased. Less than half of infants aged 7–12 months old had seropositivity and a few of toddlers had adetectable antibodies level. Thereafter, majority of children aged 1–3 years showed higher positive EV71 neutralizing antibodies level due to natural EV71 infection [15,16,17,18,19].

This study showed that the majority of reported EV71-associated HFMD cases were scattered children, which is consistent with the results of previous studies conducted in Guangzhou city, Guangdong Province and Rizhao city, Shandong Province, China [20,21]. The potential reason may be related with the following factors. In China, the scattered children are usually less than 3 years old and taken care by their grandparents with low level education and poor health-related knowledge. Moreover, those scattered children live in the crowded living places with poor sanitation.

This study result also showed that the cyclic peaks of reported number of EV71 cases occurred in children with every 11-month-age interval, e.g., 11, 23, 35, 47, 59, and 71 months age. Seropositivity of EV71 neutralizing antibodies can protect children from EV71-assocated diseases and low level seropositivity with EV71 neutralizing antibodies in children contribute to the high incidence of EV71 infection. It may be explained that children infected with EV71 could recur every 11-month interval, which is agreement with the previous study conducted in Fujian [22] and Beijing [23]. The interval time of EV71-associated HFMD recurrence was reported to be less than 12 months. Nevertheless, epidemiological data showed the interval length of recurrence of HFMD due to EV71 varied in different countries, for example, every 2–4 years in Malaysia [24], every 2–3 years in Taiwan [25], we therefore suggest to strengthen regional routine surveillance on EV71 to optimize the targeted subjects of vaccination. That complication of a primary series of EV71 vaccine before 11 months of age after birth should be essential.

Another finding of this study was the number of reported cases per square kilometer was correlated with the population density, reflecting the high reported number of cases in relative crowded cities. The similar finding was reported in other studies, which were conducted in other areas [26,27]. Poor sanitation or poor ventilation may confer to the result.

Another interesting finding in this study was the absence of October peak in 2009 and 2013. Coincidently, 2009 H1N1 and 2013 H7N9 pandemics occurred. During these periods, all Zhejiang inhabitants were advised to take particular care with personal hygiene and social distancing measures, especially, frequently hand washing and getting away from the crowed places. These two measures, together with community hygiene measures, probably resulted in reduced viral transmission chances between sick and healthy children and hence decreased number of reported EV71 cases. Various factors may affect this shift, for example, meteorological factors, population density, and herd immunity [28,29].

There are a few limitations in this study. First, this is a study based on hospital-reported cases. Those cases who did not see the doctors were excluded via NNDRS during the study period. The results in this study could be biased by this factor, which induced lower reported incidence rate per year. Secondly, there is no available data on circulating EV71 subgenogroups in the NNDRS. Therefore, future enhanced surveillance systems is recommended to focus on testing EV71 subgenogroups and further monitoring EV71 virus based on genetic and phylogenetic analysis.

## 5. Conclusions

EV71-associated HFMD remains an important public health problem in Zhejiang. In order to prevent and control EV71-associated HFMD, all susceptible children are suggested to take into account to take public health intervention, e.g., being immunized with EV71 vaccine until they reach 12–23 month, in addition to enhancing the individual hygiene measure.

## Figures and Tables

**Table 1:** Number and incidence rate of reported EV71 cases by age group, 2009–2013.

Age Group	2009		2010		2011		2012		2013		2009–2013	
(Months)	N	R	N	R	N	R	N	R	N	R	N	R
0–11	62	10	388	62.5	219	46.4	158	35.9	94	21.7	921	35.6
12–23	123	20	642	104.0	625	133.2	521	105.2	304	62.2	2215	82.5
24–35	111	17.9	547	88.3	493	104.8	401	80.9	215	44.1	1767	65.6
36–47	88	14.2	375	60.9	398	85.1	320	65.5	183	38.1	1364	51.1
48–59	47	7.5	236	38.1	168	35.7	170	34.8	106	22.1	727	27.1
60–71	16	2.5	114	18.1	92	19.3	92	20.4	37	8.4	351	13.3
≥72	22	0.1	82	0.2	90	0.2	73	0.1	38	0.1	305	0.1

N: number, R: incidence rate (1/100,000). The age group population in 2013 was defined as the denominator to calculate the standardized incidence rate.

**Table 2:** Number and incidence rate of reported EV71 cases by gender, 2009–2013.

Year	Male		Female		Ratio (Male/Female)
	N	R	N	R	
2009	297	1.1	172	0.7	1.7
2010	1497	5.7	887	3.5	1.6
2011	1318	4.7	767	2.9	1.6
2012	1098	3.9	637	2.4	1.6
2013	584	2.1	393	1.5	1.4
Total	4794	3.5	2856	2.2	1.6

N: number; R: incidence rate (1/100,000). The gender population in 2013 was defined as the denominator to calculate the standardized incidence rate.

**Table 3:** Number of reported EV71 cases by occupation, 2009–2013.

Occupation	2009 (%)	2010 (%)	2011 (%)	2012 (%)	2013 (%)	2009–2013 (%)
Scattered children	286 (61.0)	1617 (67.8)	1451 (69.6)	1195 (68.9)	675 (69.10)	5224 (68.3)
Preschool children	166 (35.4)	724 (30.4)	584 (28.0)	504 (29.0)	278 (28.5)	2256 (29.5)
School children	16 (3.4)	40 (1.7)	42 (2.0)	33 (1.9)	24 (2.5)	155 (2.0)
Others	1 (0.2)	3 (0.1)	8 (0.4)	3 (0.2)	0 (0)	15 (0.2)
Total	469 (100)	2384 (100)	2085 (100)	1735 (100)	977 (100)	7650 (100)

**Table 4:** Correlation relationship between population density and reported cases density.

City	Population/km^2^	Reported Cases/km^2^ *
Wenzhou	773.98	0.17
Ningbo	774.83	0.16
Jiaxing	1149.85	0.14
Zhoushan	778.65	0.11
Shaoxing	594.99	0.06
Huzhou	497.17	0.06
Taizhou	634.24	0.05
Quzhou	239.98	0.04
Jinhua	4900	0.04
Hangzhou	524.25	0.04
Lishui	122.38	0.03

km^2^: square kilometer; * Pearson’s correlation coefficient = 0.80, *p* = 0.003.

## Supplementary Materials

The following are available online at www.mdpi.com/1660-4601/14/1/33/s1. Table S1: Number and incidence rate of EV71 severe and dead cases by age, 2009–2013, Figure S1: Percentage of reported EV71 cases by age group, 2009–2013.

## Abbreviations

The following abbreviations are used in this manuscript:

EV71	enterovirus 71
HFMD	hand-foot-and-mouthdisease
NNDRS	national notifiable disease reporting system
PCR	polymerase chain reaction
SPSS	statistical package for the social sciences

## References

[1] McMinn P., Lindsay K., Perera D., Chan H.M., Chan K.P., Cardosa M.J. (2001). Phylogenetic analysis of enterovirus 71 strains isolated during linked epidemics in Malaysia, Singapore, and Western Australia. J. Virol..

[2] Wang J.R., Tuan Y.C., Tsai H.P., Yan J.J., Liu C.C., Su I.J. (2002). Change of major genotype of enterovirus 71 in outbreaks of hand-foot-mouth disease in Taiwan between 1998 and 2000. J. Clin. Microbiol..

[3] Chatproedprai S., Theanboonlers A., Korkong S., Thongmee C., Wananukul S., Poovorawan Y. (2010). Clinical and molecular characterization of hand-foot-and-mouth disease in Thailand, 2008–2009. Jpn. J. Infect. Dis..

[4] Tu P.V., Thao N.T., Perera D., Huu T.K., Tien N.T., Thuong T.C., How O.M., Cardosa M.J., McMinn P.C. (2007). Epidemiologic and virologic investigation of hand-foot-and-mouth disease, southern Vietnam, 2005. Emerg. Infect. Dis..

[5] Gao L.D., Hu S.X., Zhang H., Luo K.W., Liu Y.Z., Xu Q.H., Huang W., Deng Z.H., Zhou S.F., Liu F.Q. (2014). Correlation analysis of EV71 detection and case severity in hand-foot-and-mouth disease in Hunan province of China. PLoS ONE.

[6] Centers for Disease Control and Prevention Hand-Foot-and-Mouth Disease: Signs and Symptoms. http://www.cdc.gov/hand-foot-mouth/about/signs-symptoms.html.

[7] Xing W.J., Liao Q.H., Viboud C., Zhang J., Sun J.L., Wu J.T., Chang Z.R., Liu F.F., Fang V.J., Zheng Y.D. (2014). Hand-foot-and-mouth disease in China, 2008–2012: An epidemiological study. Lancet Infect. Dis..

[8] Lv H.K., Zhou Z.M., Zhang X.P., Jiang W., Zhu S.B. (2011). Analysis on epidemic situation and control of HFMD in childcare settings. Chin. Rural Health Serv. Admin..

[9] Zhu F.C., Meng F.Y., Li J.X., Mao Q.Y., Tao H., Zhang Y.T., Yao X., Chu K., Chen Q.H., Hu Y.M. (2013). Efficacy, safety, and immunology of an inactivated alum-adjuvant enterovirus 71 in children in China: A multicentre, randomized, double-blind, placebo-controlled, phase 3 trial. Lancet.

[10] Hwa S.H., Lee Y.A., Brewoo J.N., Partidos C.D., Osorio J.E. (2013). Preclinical evaluation of the immunogenicity and safety of an inactivated enterovirus 71 candidate vaccine. PLoS Negl. Trop. Dis..

[11] Li R.C., Liu L.D., Mo Z.J., Wang X.Y., Xia J.L., Liang Z.L., Zhang Y., Li Y.P., Mao Q.Y., Wang J.J. (2014). An inactivated enterovirus 71 vaccine in healthy children. N. Engl. J. Med..

[12] Zhu F.C., Xu W.B., Xia J.L., Liang Z.L., Liu Y., Zhang X.F., Tan X.J., Wang L., Mao Q.Y., Wu J.Y. (2014). Efficacy, safety, and immunogenicity of an enterovirus 71 vaccine in China. N. Engl. J. Med..

[13] Statistical Review Reports between 2009 and 2013. http://www.zj.stats.gov.cn/tjsj/tjnj/.

[14] Naing N.N. (2000). Easy way to learn standardization: Direct and indirect methods. Malays. J. Med. Sci..

[15] Ho M. (2000). Enterovirus 71: The virus, its infections and outbreaks. J. Microbiol. Immunol. Infect..

[16] Sadeharju K., Knip M., Virtanen S.M., Savilahti E., Tauriainen S., Koskela P., Akerblom H.K., Hyoty H., Finnish TRIGR Study Group (2007). Maternal antibodies in breast milk protect the child from enterovirus infections. Pediatrics.

[17] Witso E., Cinek O., Aldrin M., Grinde B., Rasmussen T., Wetlesen T., Ronningen K.S. (2010). Predictors of sub-clinical enterovirus infections in infants: A prospective cohort study. Int. J. Epidemiol..

[18] Zhu F.C., Liang Z.L., Meng F.Y., Zeng Y., Mao Q.Y., Chu K., Song X.F., Yao X., Li J.X., Ji H. (2012). Retrospective study of the incidence of HFMD and seroepidemiology of antibodies against EV71 and CoxA16 in prenatal women and their infants. PLoS ONE.

[19] Chia M.Y., Chiang P.S., Chung W.Y., Luo S.T., Lee M.S. (2014). Epidemiology of enterovirus 71 infections in Taiwan. Pediatr. Neonatol..

[20] Li T.G., Yang Z.C., Liu X.Y., Kang Y., Wang M. (2014). Hand-food-and-mouth disease epidemiological status and relationship with meteorological variables in Guangzhou, southern China, 2008–2012. Rev. Inst. Med. Trop. Sao Paulo.

[21] Wu H.X., Wang H.C., Wang Q.Z., Xin Q.H., Lin H.L. (2014). The effect of meteorological factors on adolescent hand-foot-and-mouth disease and associated effect modifiers. Glob. Health Act..

[22] Xie Z.H., Zhang Y.Z., Yan Y.S., Hong R.T., Wang L.L. (2013). Preliminary study on reccurance with viruses causing hand foot and mouth disease. Dis. Surveill..

[23] Cao Z.D., Zeng D.J., Wang Q.Y., Zheng X.L., Wang F.Y. (2010). An epidemiological analysis of the Beijing 2008 Hand-Foot-Mouth epidemic. Chin. Sci. Bull..

[24] Chua K.B., Kasri A.R. (2011). Hand foot and mouth disease due to enterovirus 71 in Malaysia. Virol. Sin..

[25] Huang S.W., Hus Y.W., Smith D.J., Kiang D., Tsai H.P., Lin K.H., Wang S.M., Liu C.C., Su I.J. (2009). Reemergence of Enterovirus 71 in 2008 in Taiwan: Dynamics of genetic and antigenic evolution from 1998 to 2008. J. Clin. Microbiol..

[26] Wang Q., Wang Z.J. (2010). Epidemiology of hand-foot-and-mouth disease in China, 2008. Dis. Surveill..

[27] Lin H.L., Zou H., Wang Q.Z., Liu C.X., Lang L.L., Hou X.X., Li Z.J. (2013). Short-term effect of EI Nino-southern oscillation on pediatric hand, foot and mouth disease in Shenzhen, China. PLoS ONE.

[28] Hii Y.L., Rocklov J., Ng N. (2011). Short term effects of weather on hand, foot and mouth disease. PLoS ONE.

[29] Ooi M.H., Wong S.C., Podin Y., Akin W., Sel S., Mohan A., Chieng C.H., Perera D., Clear D., Wong D. (2007). Human enterovirus 71 disease in Sarawak, Malaysia: A prospective clinical, virological, and molecular epidemiological study. Clin. Infect. Dis..

