# Supplementary material for: Epidemiologic Features of Enterovirus 71-Associated Hand-Foot-and-Mouth Disease from 2009 to 2013 in Zhejiang, China
Source: Int J Environ Res Public Health. 2016 Dec 30;14(1):33. doi: 10.3390/ijerph14010033 (PMC5295284; doi:10.3390/ijerph14010033)
Supplement: Supplementary file 1 [file ijerph-14-00033-s001.pdf]

Supplementary Materials: Epidemiologic Features of Enterovirus 71-Associated Hand-Foot-and-Mouth Disease from 2009 to 2013 in Zhejiang, China

Zhifang Wang, Huakun Lv, Wenming Zhu, Zhe Mo, Guangming Mao, Xiaofeng Wang, Xiaoming Lou and Yongdi Chen

Table S1. Number and incidence rate of EV71 severe and dead cases by age, 2009–2013.

Age Group (ms)	2009		2010		2011		2012		2013		2009–2013	
Severe Cases	N	R	N	R	N	R	N	R	N	R	N	R
0–11	24	3.9	225	36.2	18	3.8	10	2.3	4	0.9	281	10.9
12–23	23	3.7	185	30	72	15.3	26	5.3	8	1.6	314	11.7
24–35	16	2.6	92	14.9	40	8.5	5	1.0	2	0.4	155	5.8
36–47	10	1.6	44	7.1	30	6.4	9	1.8	1	0.2	94	3.5
48–59	2	0.3	15	2.4	12	2.5	3	0.6	5	1.0	37	1.4
60–71	1	0.2	12	1.9	2	0.4	2	0.4	0	0	17	0.6
≥72	0	0	5	0	5	0	1	0	0	0	11	0
Dead Cases												
0–11	4	0.6	13	2.1	7	1.5	8	1.8	2	0.5	34	1.3
12–23	2	0.3	11	1.8	6	1.3	7	1.4	0	0	26	1.0
24–35	2	0.3	5	0.8	7	1.5	1	0.2	0	0	15	0.6
36–47	0	0	0	0	1	0.2	0	0	0	0	1	0
48–59	0	0	0	0	0	0	0	0	0	0	0	0
60–71	0	0	0	0	0	0	0	0	0	0	0	0
≥72	0	0	0	0	0	0	0	0	0	0	0	0

N: number; ms: months; R: incidence rate (1/100,000). The age group population in 2013 was defined as the denominator to calculate the standardized incidence rate.

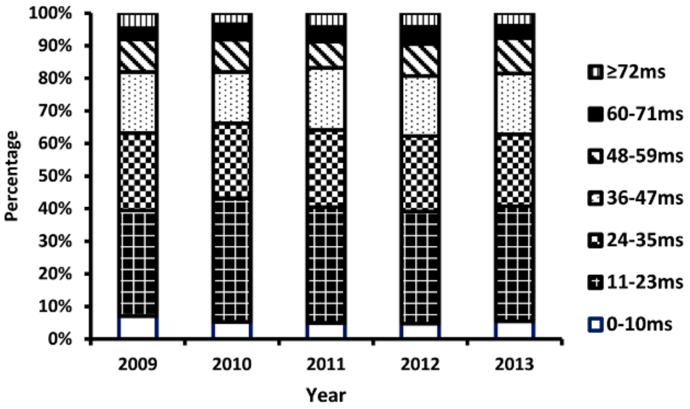

Figure S1. Percentage of reported EV71 cases by age group, 2009–2013.

© 2016 by the authors. Submitted for possible open access publication under the terms and conditions of the Creative Commons Attribution (CC-BY) license (http://creativecommons.org/licenses/by/4.0/).
